# MOXO-d-CPT in Adults: Useful or Premature? An Exploratory Study on Clinical Relevance

**DOI:** 10.1192/j.eurpsy.2026.12013

**Published:** 2026-07-31

**Authors:** A. Alp, B. Demir

**Affiliations:** 1 Psychiatry, Gazi Mustafa Kemal State Hospital; 2 Psychiatry, Hacettepe University Faculty of Medicine, Ankara, Türkiye

## Abstract

**Introduction:**

The MOXO-d-CPT (MOXO-Distracted Continuous Performance Test) is a computerized assessment tool used to support the diagnosis of Attention Deficit Hyperactivity Disorder (ADHD) in both children and adults. The test consists of four sub-indices: attention, timing, impulsivity, and hyperactivity. MOXO-d-CPT utilizes playing cards instead of alphanumeric stimuli, and incorporates distractors commonly encountered in daily life, such as crying babies or arguing parents. Although the test has demonstrated 85% validity and reliability in children, no established validation or reliability studies exist for adult populations. This lack of validation may contribute to the test’s limited clinical and research use adult populations.

**Objectives:**

This study aimed to examine the associations between MOXO-d-CPT performance and ADHD trait levels, as well as performance on the Stroop and Trail Making Tests (TMT), in a healthy adult sample.

**Methods:**

A total of 223 healthy university students participated in the study. All participants completed the MOXO-d-CPT, the Stroop test, and the Trail Making Test. ADHD trait levels were assessed using the Turgay Adult ADHD Rating Scale, which is based on DSM-5 criteria and has been validated in Turkish adult populations. Correlation analyses were conducted using Spearman’s rho.

**Results:**

No significant relationship was found between the MOXO timing index and clinical ADHD symptoms. Among the MOXO indices, the most consistent finding emerged in the impulsivity domain. However, even the strongest association, between MOXO impulsivity and the total ADHD score, reached only a moderate level (r = - .450, p < .001). The attention and hyperactivity indices of MOXO showed weak correlations with ADHD symptom levels. Furthermore, no meaningful or at least moderate correlations were observed between MOXO indices and Stroop or TMT performance measures, including total Stroop duration, total errors, or correction counts.

**Conclusions:**

The integration of realistic distractors, a practical 18-minute application time, and the innovative inclusion of a separate timing index are valuable contributions to the development of continuous performance tests. However, the current findings emphasize the urgent need for comprehensive validity and reliability studies before MOXO-d-CPT can be confidently recommended for ADHD assessment in adult clinical settings.

**Disclosure of Interest:**

None Declared

